# Analysis of Shielding Effectiveness against Electromagnetic Interference (EMI) for Metal-Coated Polymeric Materials

**DOI:** 10.3390/polym15081911

**Published:** 2023-04-16

**Authors:** Seyed Jamaleddin Mostafavi Yazdi, Andrej Lisitski, Seongchan Pack, Huseyin R. Hiziroglu, Javad Baqersad

**Affiliations:** 1 NVH & Experimental Mechanics Laboratory, Department of Mechanical Engineering, Kettering University, 1700 University Ave, Flint, MI 48504, USA; 2 Global Product Development at Global Technical Center, General Motors, Warren, MI 48340, USA; 3 Department of Electrical & Computer Engineering, Kettering University, 1700 University Ave, Flint, MI 48504, USA

**Keywords:** EMI, coating, EMI shielding, shielding effectiveness, polymer, metal

## Abstract

Lightweight materials, such as polymers and composites, are increasingly used in the automotive and aerospace industries. Recently, there has been an increase in the use of these materials, especially in electric vehicles. However, these materials cannot shield sensitive electronics from electromagnetic interference (EMI). The current work investigates the EMI performance of these lightweight materials using an experimental setup based on the ASTM D4935-99 standard and EMI simulation using the ANSYS HFSS. This work studies how metal coating from zinc and aluminum bronze can improve the shielding performance of polymer-based materials, such as polyphenylene sulfide (PPS), polyetheretherketone (PEEK), and polyphthalamide (PPA). Based on the findings of this study, a thin coating (50 μm) of Zn on the surface of PPS and a thin coating of 5 μm and 10 μm of Al-Bronze, respectively, on the surface of PEEK and PPA have indicated an increase in the shielding effectiveness (SE) when subjected to EMI. The shielding effectiveness significantly increased from 7 dB for the uncoated polymer to approximately 40 dB at low frequencies and up to approximately 60 dB at high frequencies for coated polymers. Finally, various approaches are recommended for improving the SE of polymeric materials under the influence of EMI.

## 1. Introduction

Novel composites have been preferred in the automotive and aerospace industries because of their lightweight and high stiffness [1]. As the number of electric vehicles (EVs) is rapidly growing, polymer-based composites are increasingly used in a wide variety of applications in the automotive industry [2]. These modern vehicles rely heavily on sophisticated electronic devices and systems operating at high frequencies ranging from several kHz to several GHz. Most of these electronic devices and systems facilitate the healthy operation of a vehicle in conjunction with sensors that acquire various types of data and convert them into appropriate electrical signals. In addition, the safety of the driver and passengers in these modern vehicles is highly dependent on the integrity of the acquired signal. The operation of these high-frequency electronic systems should not be adversely affected, and the integrity of the electrical signal should not be compromised by the interference due to the fact of electromagnetic noise that could be present in the vicinity. Otherwise, the consequences could possibly be catastrophic. Thus, the electronic system must be well shielded against electromagnetic noise for its proper operation. This problem has been traditionally solved by incorporating highly conductive materials, such as copper, as a shield to house the electronic system. The coating of copper nanoparticles on flexible substrates can significantly improve the electromagnetic shielding’s effectiveness [3]. In modern vehicles, if the traditional method of shielding with metals is used, the extra mass will become significant, since the number of electronic systems is many multiples of those in conventional vehicles. The solution to this problem could possibly be the utilization of polymer-based composites. However, because the base polymer is predominantly a dielectric material with poor shielding effectiveness (SE), the signal integrity of a delicate electronic sensor system becomes vulnerable to EMI when housed in a polymer-based composite, making it unacceptable in modern vehicles. Nevertheless, provided that the SE is evaluated by employing accurate techniques, the SE of the polymer composites can be significantly improved by doping the base material with conducting particles or coating it with a thin film of appropriate conducting material. For example, A polymer nanocomposite, such as ABS loaded with multi-walled carbon nanotubes (CNTs), has a higher shielding effectiveness than neat ABS [4]. Magnetic nanoparticles (NPs) are known for their superior EMI shielding; combining them with CNTs can enhance the effective shielding [5]. Together, these results provide important insight into a polymer material with a metal coating.

To determine the SE of a certain material, experimental techniques with a vector network analyzer have been employed in previous studies [6]. The SE of such materials, including flexible liquid–metal composites [7], shape memory polymer composites [8], fiber-reinforced cementitious composites [9], integrated metal mesh/TPU/CIP composites [10], were investigated by other researchers.

Although metals are used for shielding because of their high conductivity [11] to obstruct the emission of electromagnetic waves, their heavy mass is a challenge for light vehicle applications [12]. Markham [13] studied different materials for their SE using the ASTM-4935-83 standard and found that carbon-based filler had the best SE for portable electronic devices such as notebooks. In a review of the EMI SE of carbon materials, Chung [14] concluded that carbon filaments electroplated with nickel were more effective than other carbon composites for the EMI SE. The silver (Ag) layer on the coating of the fabric complex could increase the total EMI SE by 3 dB up to the frequency of 0.5 GHz [15]. Yuping et al. [16] investigated the EMI SE of different composites made of silicone rubber and found that composites with PAN-HCl loading had a high SE.

While Chen et al. [17] investigated the effect of the Y content on the mechanical properties of Mg-Zn-Y-Zr alloy, they also found that increasing the Y content could significantly improve the EMI SE of the alloy. Kasgoz et al. [18] investigated the microstructure and EMI SE of TPU-CNF and found that a composite material with a thickness of 2 mm can efficiently block electromagnetic waves in the 8–12 GHz frequency range. The SE of graphite-reinforced carbon composite was measured using a vector network analyzer at 38.6 dB [19]. A new SE tester was manufactured according to the ASTM D4935 to measure the EMI SE of conductive polymer composites at a frequency lower than 3 GHz [20]. The effects of the fiber volume ratio and number of composite layers on the SE of laminated epoxy composites were investigated by Munalli et al. [21]. They found that four composite layers can attenuate the electromagnetic field by more than 99.9%. The fiber volume ratio can affect the reflection of composite material more than the composite layers. The EMI SE of different fiber sizes and lengths was optimized for carbon fiber-reinforced cementitious composite [22]. Wanasinghe et al. [22] found that a 0.7% volume fraction of fiber with a length of 12 mm can reach an SE of 40–60 dB.

As seen in the above literature review, despite extensive studies conducted to quantify the EMI SE, there is little or no information from studying the EMI shielding of polymer-based materials and how their EMI can be improved using highly conductive metal coating technology. Therefore, the objective of this study was to investigate the influence of the metal coating on the EMI SE of polymer-based material using experimental techniques, as well as theoretical simulation.

## 2. Experiments

### 2.1. Materials

In this work, we studied the effects of different metal coatings, such as aluminum, aluminum bronze, and zinc, on polymer-based materials. All glass fiber-reinforced PPA, PPS, and PEEK were provided from Solvay Specialty Polymers. They were molded through an injection molding process. Zinc or aluminum or Al-bronze coatings were applied by a thermal spray process. These polymeric materials significantly improve noise and vibration performance when coated with metals.

**Figure 1:**
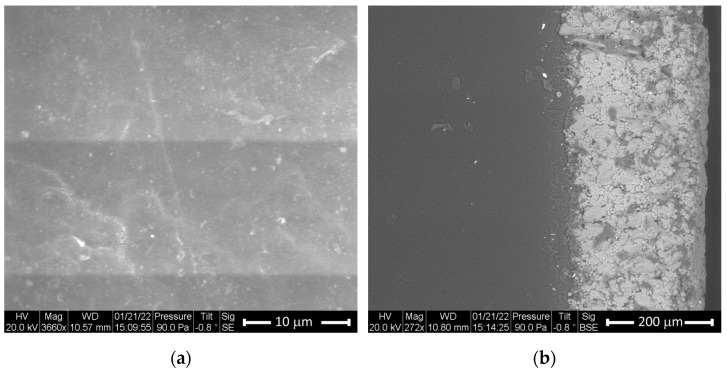
ESEM images: (**a**) uncoated PEEK surface; (**b**) PEEK with aluminum coating.

The microstructures in the polymer and the coating material were studied using an environmental scanning electron microscope (ESEM). A Philips FE1 Quanta 200 ESEM was utilized to image the microstructure of polyether ether ketone (PEEK) and PEEK coated with aluminum. As shown in Figure 1a, the ESEM secondary micrograph of the PEEK sample shows the dark reflective regions. Figure 1b shows a dense homogenous microstructure with no significant damage to PEEK, with a relatively uniform surface. The homogenous microstructure of the PEEK allowed us to create a homogenous finite element model for the theoretical calculations.

**Figure 2:**
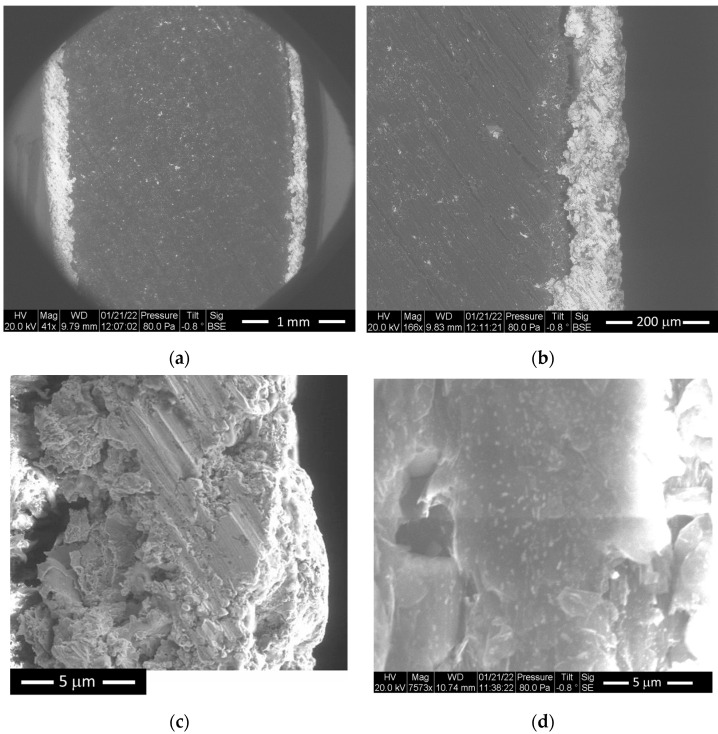
The ESEM of PPS with zinc coating: (**a**) backscattered (41×); (**b**) backscattered (166×); (**c**) zinc coating backscattered (862×); (**d**) PPS with 7573×.

In addition, Figure 1b illustrates the backscattered image of the PEEK coated with aluminum. As can be seen in this figure, the aluminum coating covered the entire surface and presents a less homogenous microstructure than the substrate. Figure 2 shows the backscattered images of zinc-coated polyphenylene sulfide (PPS) with different SEM zooms.

**Figure 3:**
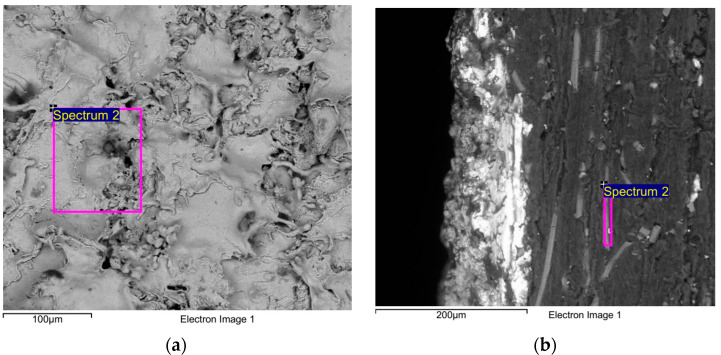
EDAX analysis: (**a**) zinc; (**b**) PPS materials.

In addition, we performed the energy-dispersive X-ray analysis (EDXA or EDAX) analysis for the zinc and PPS materials, as shown in Figure 3. The EDXA analysis shows the weight percentage of the zinc and sulfur in PPS at 77% and 28.18%, respectively. The results of the EDAX analysis are shown in Figures S1 and S2 (see Supplementary Materials).

### 2.2. Experimental Setup

**Figure 4:**
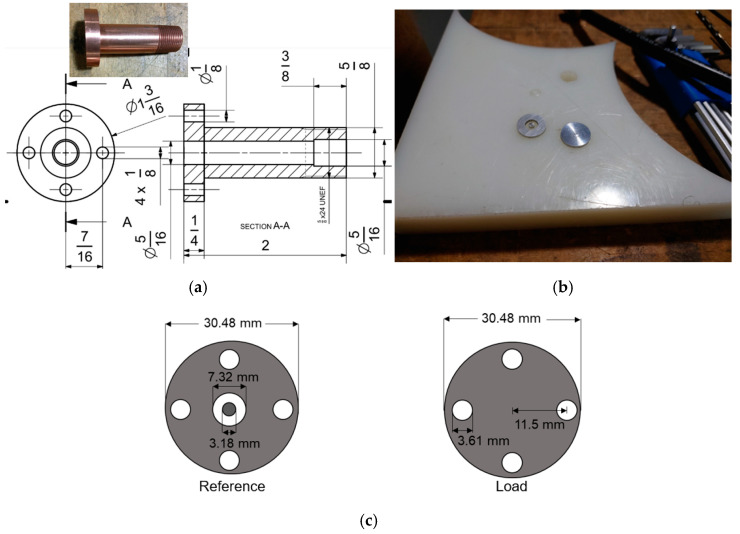

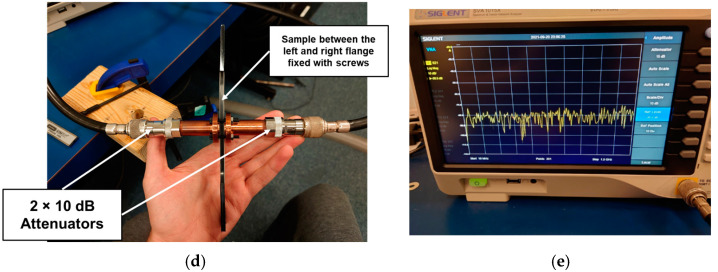
(**a**) Dimensions of one-half of the coaxial fixture used in the experiments; (**b**) cut sample materials with specific geometry; (**c**) dimensions of the reference and load samples; (**d**) coaxial fixture made of copper with two terminals connected to the vector network analyzer with the test sample between the two center flanges, according to the ASTM 4935-99; (**e**) vector network analyzer.

Many researchers have utilized the ASTM D4935-99 standard [23] to quantify the SE of a material. However, this standard has constraints such as the size of the samples and the frequency range. A small-scale, coaxial waveguide made of copper, shown in Figure 4a, was designed to have a 50 Ω characteristic impedance and manufactured in the laboratory, as shown in the photograph in Figure 4c, to comply with the sample size requirement of the ASTM D4935-99. A similar fixture by Vasquez et al. [24] has also shown promising results in measuring the EMI SE of samples up to 1.5 GHz.

A vector network analyzer, Siglent SVA1015X, shown in Figure 4d, with a frequency range from 9 kHz to 1.5 GHz, was used to quantify the EMI SE of the samples in a frequency range of 150 kHz–1.5 GHz, since this frequency range is sufficient for many industrial applications. In this method, the measurements with planar samples, when excited by a plane, far-field electromagnetic (EM) wave, are valid in the sample frequency range. The GM material group carefully prepared the materials and cut them into the specific geometry, as indicated in Figure 4b.

Two 10 dB attenuators were used at each side of the sample holder to increase the dynamic range of the vector network analyzer and to protect the receiving sensor against high voltages, as indicated in Figure 4c.

The SE value was calculated using S_21_ and S_11_ scattering parameters (S-parameters) measured by the vector network analyzer.

The total, absorption, and reflection SE are calculated using Equations (1)–(5):

(1) $$R={\left|S_{11}\right|}^{2}$$

(2) $$T={\left|S_{21}\right|}^{2}$$

(3) $${SE}_{T}=10\;log\;(1/T)$$

(4) $${SE}_{R}=10\;log\;[1/(1-R)]$$

(5) $${SE}_{A}=10\;log\;[(1-R)/T]$$

where R is the reflection coefficient, T is the transmission coefficient, S_11_ is the voltage reflection coefficient of the input port when the output port is matched, S_21_ is the reverse voltage gain, S_22_ is the voltage reflection coefficient of the output port, when the input port is matched, SE_T_ is the total shielding effectiveness, SE_A_ is the absorption shielding effectiveness, and SE_R_ is the reflection shielding effectiveness [23].

**Figure 5:**
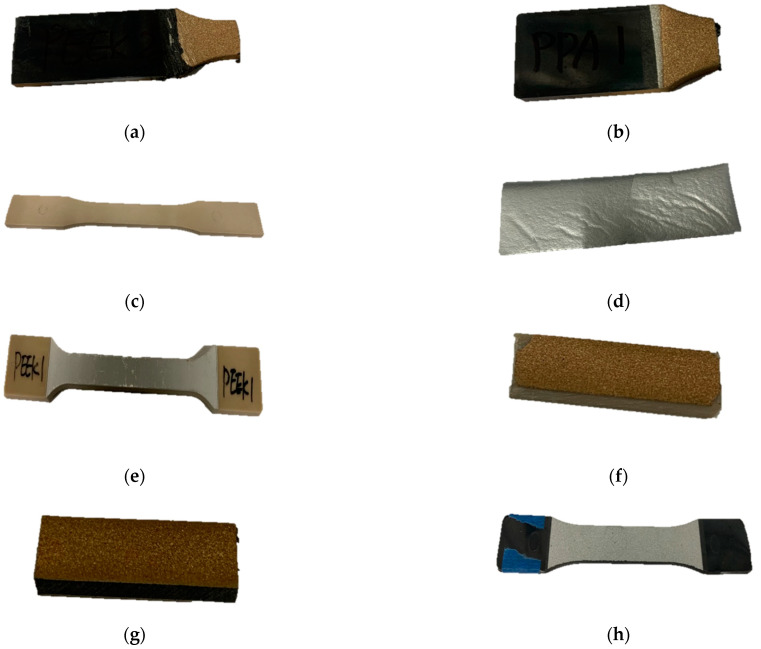
The samples were made of (**a**) PEEK; (**b**) PPA; (**c**) PPS; (**d**) zinc; (**e**) PEEK with aluminum coating; (**f**) PEEK with aluminum bronze coating; (**g**) PPA with aluminum bronze coating; (**h**) PPS with zinc coating.

Different polymers, such as PEEK, polyphenylene sulfide (PPS), and polyphthalamide (PPA), with metal coatings made of aluminum, aluminum bronze, and zinc, whose photographs are presented in Figure 5, were analyzed and simulated for the EMI SE. The polymer-based materials, PEEK, PPA, and PPS, are shown in Figure 5a–c, respectively. In addition, the zinc coating is illustrated in Figure 5d. Aluminum bronze and zinc coatings covered the surface of the polymer-based material and zinc using a thermal spray process. Figure 5e–h show the metal coatings on polymer-based materials. The samples were received in a dog bone shape. We performed the noise vibration and harshness (NVH) tests in addition to the EMI ones. The NVH results will be published as a separate research paper.

Table 1 shows the material properties of different samples utilized for the ANSYS HFSS simulations.

### 2.3. Simulation Using ANSYS HFSS

**Figure 6:**
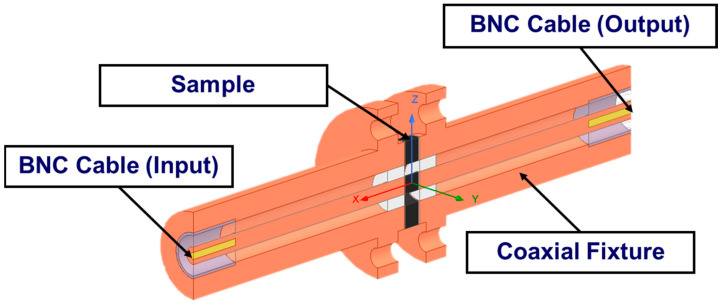
A coaxial model with a sample to simulate the EMI SE in ANSYS HFSS.

ANSYS HFSS 2021 R2 was used as a simulation tool to predict the SE of the sample materials and to compare the results of the simulation with the experimental measurements. The two ports mentioned (i.e., input and output ports), with an impedance of 50 µm, as shown in Figure 6, were connected to the vector network analyzer. The input port generated the electric field excitation, and the output port received the electromagnetic waves. The size of the circular waveguide was based on the ASTM 4935-99 D standard, as illustrated in Figure 4. Only half of the coaxial fixture was modeled due to the axial symmetry to reduce the computational time in the simulation. The BNC connectors of the cables at the input and output ports are illustrated in Figure 6. The parameters of the coating materials are shown in Table S1 (see Supplementary Materials). The mesh quality of the sample and fixture was considered a default value in the ANSYS HFSS. The frequency range was selected from 10 kHz to 1.5 GHz as the frequency sweep. All the calculations assumed that the materials were homogenous with linear properties. In addition, the surface quality of the fixture was considered to be smooth in the calculations.

## 3. Results and Discussion

### ANSYS Simulation

**Figure 7:**
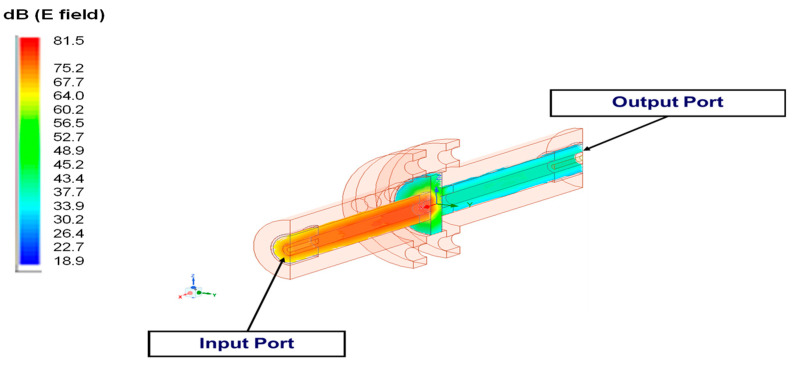
Simulation of the E-Field at 1.05 GHz for the PPS sample in the EMI fixture.

**Figure 8:**
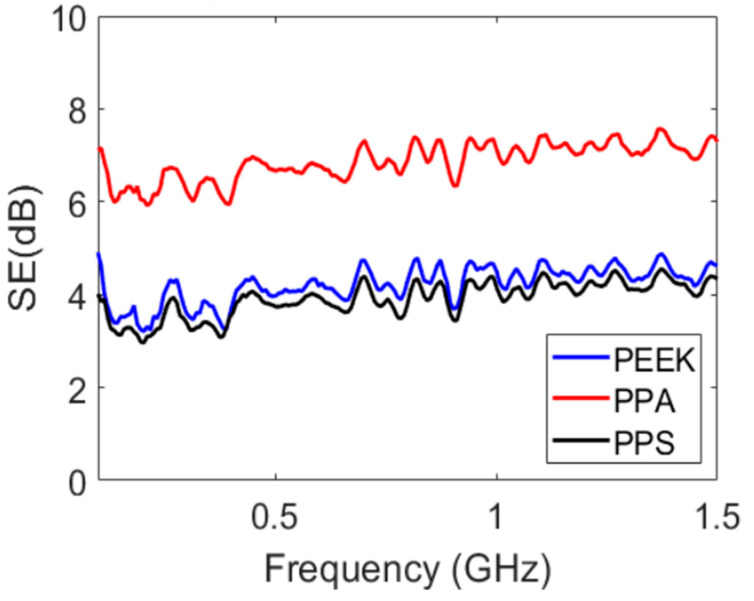
Measured total shielding effectiveness of three polymer materials.

With the ANSYS HFSS simulation, the input port of the coaxial fixture was excited by an electromagnetic wave at 1.05 GHz incident to the PPS sample placed in the fixture, as illustrated in Figure 6, similar to what was conducted in the experiments. Figure 7 presents the distribution of the E-field of the electromagnetic wave in dB at 1.05 GHz for the PPS material. It was apparent from this electric field distribution that there existed an attenuation on the electromagnetic wave as the wave traveled through the PPS sample, as evident from the weak electric field distribution at the output port of the coaxial fixture compared to its input port, as shown in Figure 7. This attenuation amounted to an SE of approximately 4 dB, as can be observed in Figure 8 for PPS. In addition, the simulation of the E-Field at 1.05 GHz for the PPA sample in the EMI fixture is shown in Figure S4 (see Supplementary Materials).

**Figure 9:**
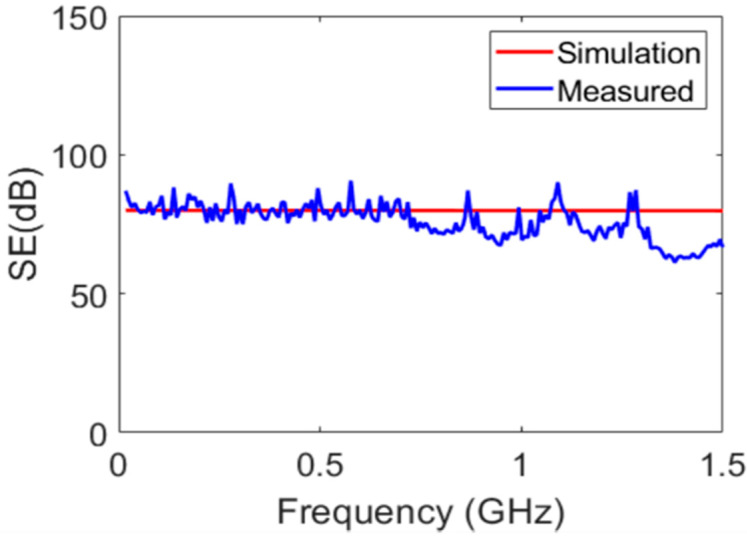
Comparison of the zinc EMI SE using an ANSYS simulation and experimental measurements.

Both the experimental SE results and the SE results from the simulation for Zn are presented in Figure 9. It is apparent that Zn had a much higher SE value than PPS, as expected.

**Figure 10:**
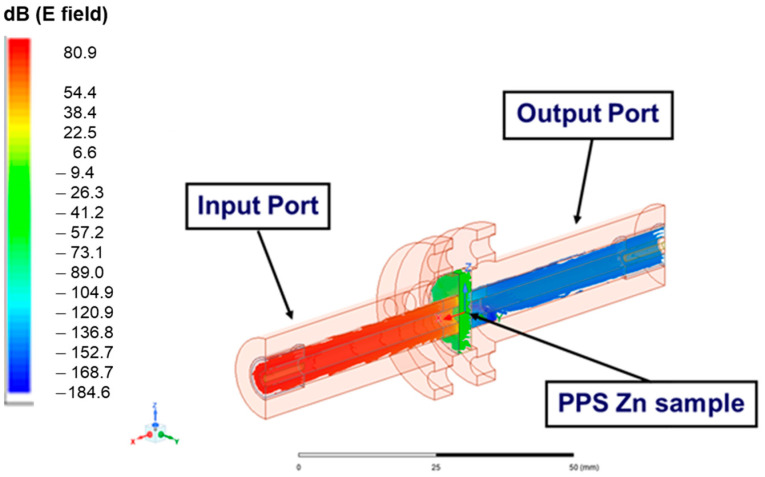
E-field for the PPS zinc sample.

Figure 10 shows the electric field distribution for a PPS Zn sample, which blocked the EM wave through the fixture. In this case, a 50 μm zinc coating was applied to the top and bottom layers of the PPS, which led to an overall increase in the conductivity of the sample. As can be seen in Figure 10, the distribution of the electric field was the highest for the wave incident right before the PPS Zn at the input port of the fixture, while it diminishes substantially on the layer facing its output port.

**Figure 11:**
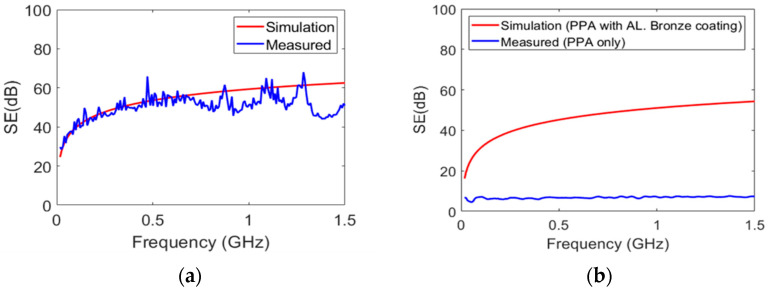
The EMI SE: (**a**) PPS with Zn coating; (**b**) PPA without and with aluminum bronze coating (5 µm).

As a result of the ANSYS HFFL simulation, the SE of the PPS Zn increased to 20 dB at 10 kHz, reaching approximately 60 dB at 1.5 GHz as indicated in Figure 11. Figure 11a also presents a comparison of the measured and simulated SE of a PPS Zn sample. The simulation results followed the experimental results closely with a small error margin up to the frequency of 0.7 GHz. The discrepancy between the measured and simulated results, nevertheless, was less than 9.2% at higher frequencies. In the context of the simulation, the material properties were considered to be linear, and the surface properties inside the fixture were assumed to be smooth. These assumptions might possibly cause a slight discrepancy between the experimental and simulation results. Figure 11b reveals that when PPA is subjected to electromagnetic waves, its SE is approximately 7 dB, which is quite low.

**Figure 12:**
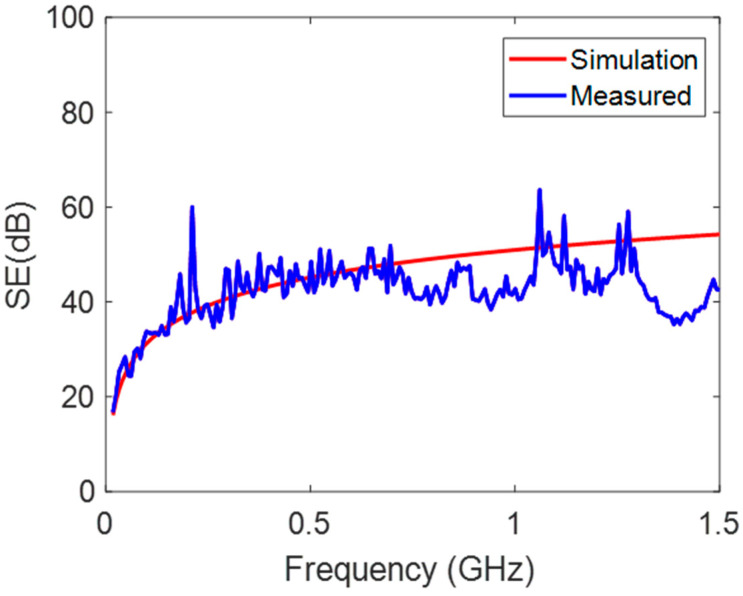
EMI SE of the PPA-based material with an aluminum bronze coating.

From the same figure, however, it is apparent when PPA is coated with aluminum bronze having a thickness of 5 μm, the SE of the coated PPA can increase to an average of approximately 40 dB, even at low frequencies, while in the frequency range from 10 kHz to 1.5 GHz, the SE in the coated PPA reached to a value of 57 dB. The increase in SE was essentially due to the increase in the conductivity of the aluminum bronze-coated PPA. There was also good agreement with a discrepancy of 7.6% between the experimental and theoretical results of the SE in the aluminum bronze-coated PPA, as shown in Figure 12.

**Figure 13:**
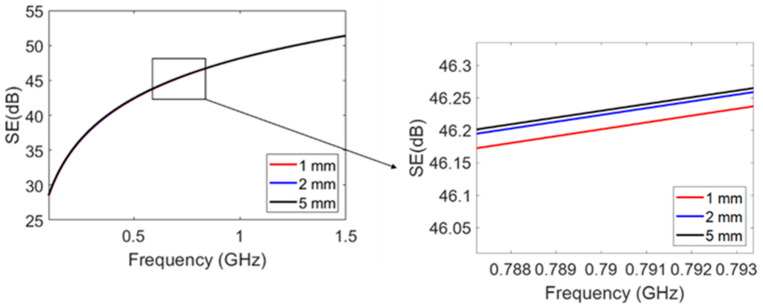
Comparison of different thicknesses of coating layers on the EMI SE of PPA with an aluminum bronze coating (simulation using ANSYS HFSS).

In addition, theoretically investigated within the scope of this study was the influence of the thickness of the coating on the SE of the polymeric material. PPA was coated with aluminum bronze with thicknesses of 1 mm, 2 mm, and 5 mm. Although with the increase in the thickness of the aluminum bronze coating, the conductivity of the coated PPA increased almost five times, only an insignificant increase in SE was observed, as presented in Figure 13. Thus, any thickness of metal coating would result in an excellent SE performance.

**Figure 14:**
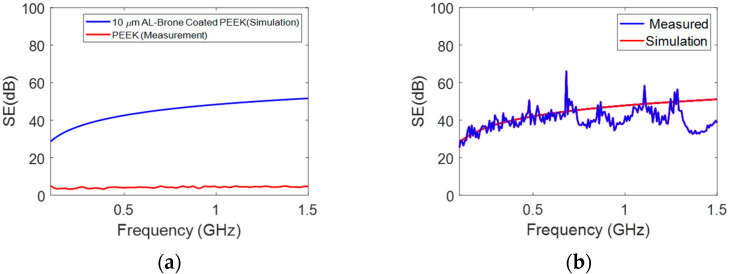
(**a**) The comparison between the SE of PEEK and PEEK with 10 µm aluminum bronze coating; (**b**) EMI SE of PEEK-based material with aluminum coating.

As shown in Figure 14a, adding a thin layer (10 µm) of Al-bronze can considerably increase the EMI SE to more than 20 dB. It is apparent from Figure 14b that Al-bronze coating on the PEEK as a base material can increase the EMI SE from about 30 dB to approximately 50 dB when the frequency is increased from 10 kHz to 1.5 GHz, respectively.

As seen from the above results, the SE of lightweight, composite polymeric materials can be significantly raised to acceptable levels for mitigating EMI by coating their surfaces appropriately with materials having high conductivity. However, it should be noted that there are challenges in the manufacturing process of the thin coating. Therefore, necessary precautions should be considered when the thin coating is used.

## 4. Conclusions

Lightweight composite materials have been preferred in the automotive and aerospace industries due to the fact of their high performance. In this study, the SE of various polymeric materials was investigated against EMI experimentally using VNA and theoretically using ANSYS HFSS software, since these materials could be used to house critical, high-frequency electronic devices and on-board systems. PEEK, PPS, and PPA were the three polymeric materials considered in this study. Although these materials have indicated SE as we evaluated experimentally within a frequency range of 10 kHz–1.5 GHz, the values were inferior, as expected, and not at acceptable levels at all. As a possible solution, PPS was coated with a 50 μm thickness of Zn, and PPA and PEEK were coated, respectively, with 5 μm and 10 μm thicknesses Al-bronze to increase the SE of these polymers. Our findings indicate a substantial increase in the SE of these three kinds of coated polymers, typically from 7 dB for the uncoated polymer to approximately 40 dB at low frequencies and up to approximately 60 dB at high frequencies for coated polymers. Therefore, these coated polymeric materials could be used in industrial applications against EMI. However, the challenges in the manufacturing process and proper coating coverage should always be kept in mind when a thin coating is used. Further studies on the variation of the coating surface roughness [25], effects of the structure relaxation and surface oxidation [26], and microstructure and tribological properties [27,28,29] on the EMI SE will need to be undertaken.

Another important conclusion of this study is the accuracy of the theoretical results for SE calculations. It was found that the discrepancy between the experimental and theoretical results was always less than 10% within the frequency range considered in this study. Therefore, with appropriate tools, theoretical calculations might be very helpful in predicting the SE for these polymers during the design stage without the construction of expensive test setups.

## Figures and Tables

**Table 1:** The electrical properties of the samples.

Material	Conductivity (S/M)	Dielectric Constant	Thickness (MM)
Aluminum	3.8 × 10^7^	1	0.250
Zinc	1.2 × 10^7^	1	0.050
PPA GF	1.5 × 10^−13^	4.3	3.960
PPS	4.5 × 10^−14^	3.0	3.290
PEEK	4.9 × 10^−14^	3.3	4.070
Al-Bronze	1.368 × 10^7^	1	0.200

## Data Availability

The data presented in this study are available on request from the corresponding author.

## Supplementary Materials

The following supporting information can be downloaded at: https://www.mdpi.com/article/10.3390/polym15081911/s1, Figure S1: The EDAX analysis of Zinc; Figure S2: The EDAX analysis of PPS; Figure S3: The XRD analysis of Zn_PPS; Figure S4: Simulation of E-Field at 1.05 GHz for PPA sample in the EMI fixture; Table S1: EMI simulation parameters of coatings.
