# Supplementary material for: Analysis of Shielding Effectiveness against Electromagnetic Interference (EMI) for Metal-Coated Polymeric Materials
Source: Polymers (Basel). 2023 Apr 16;15(8):1911. doi: 10.3390/polym15081911 (PMC10143063; doi:10.3390/polym15081911)
Supplement: Supplementary file 1 [file polymers-15-01911-s001.zip › polymers-2274462-supplementary.pdf]

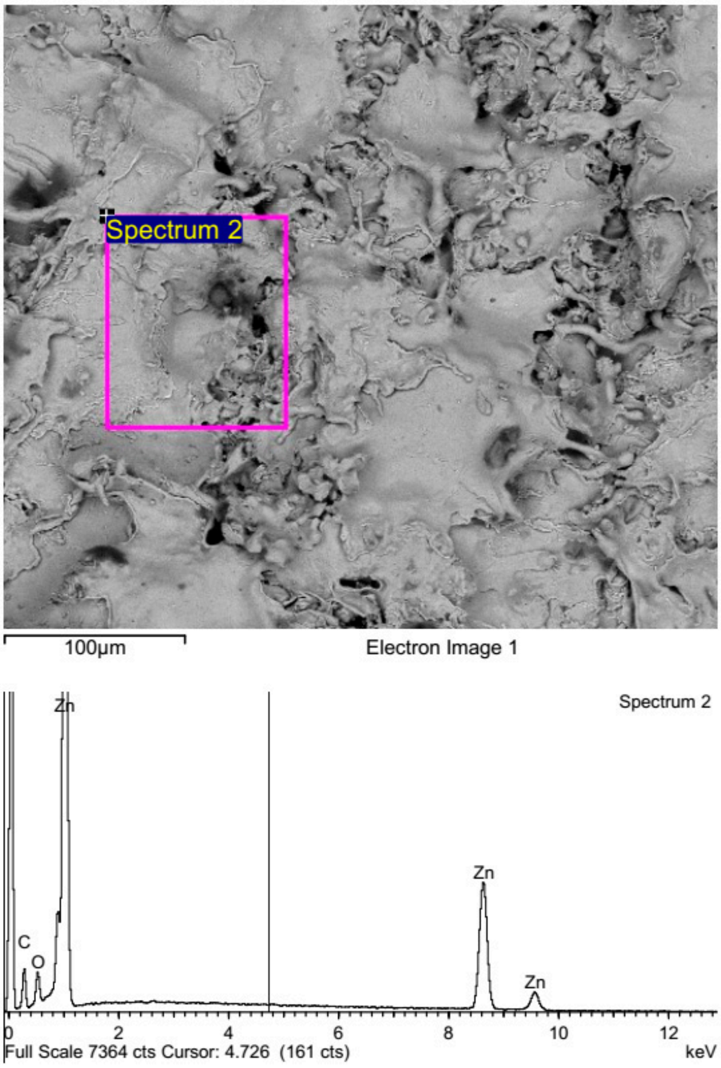

Spectrum processing :
No peaks omitted

Processing option : All elements analyzed (Normalised)
Number of iterations = 3

Standard :
C CaCO3 1-Jun-1999 12:00 AM
O SiO2 1-Jun-1999 12:00 AM
Zn Zn 1-Jun-1999 12:00 AM

Element	Weight%	Atomic%
C K	18.49	51.59
O K	4.18	8.76
Zn K	77.33	39.65
Totals	100.00	

Figure S1. The EDAX analysis of Zinc.

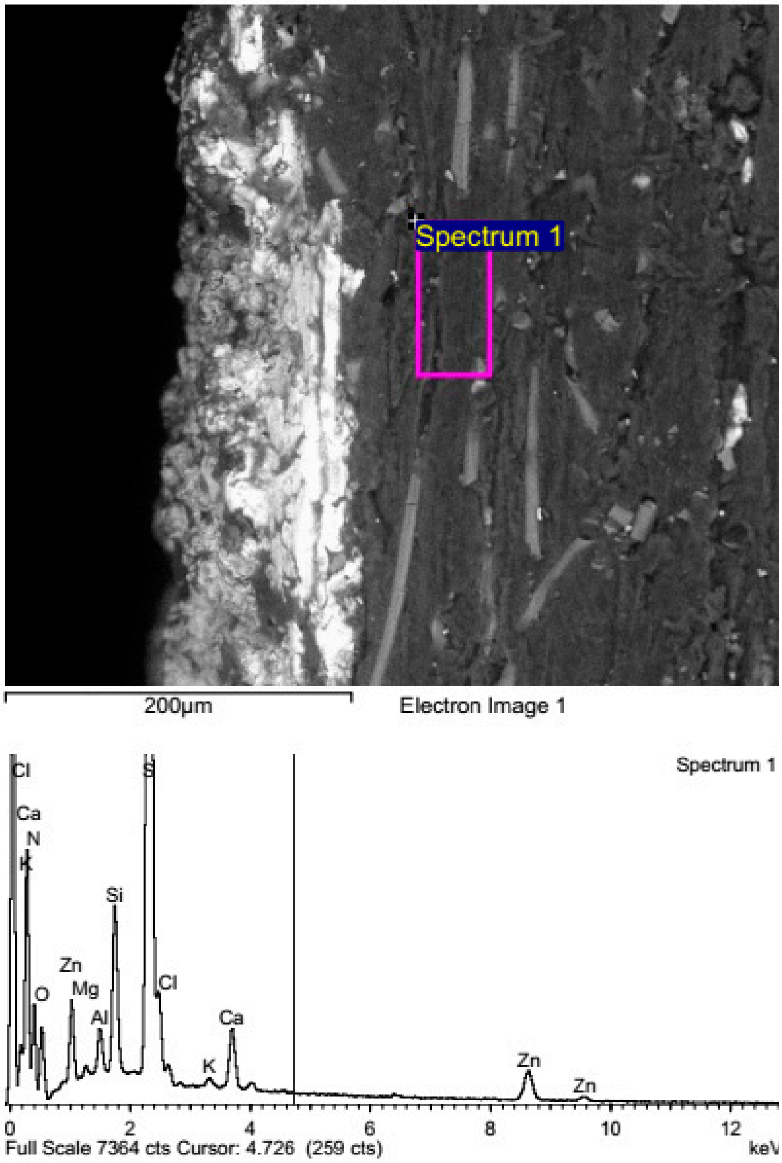

Spectrum processing :
Peak possibly omitted : 6.421 keV

Processing option : All elements analyzed (Normalised)
Number of iterations = 5

Standard :
N Not defined 1-Jun-1999 12:00 AM
O SiO2 1-Jun-1999 12:00 AM
Mg MgO 1-Jun-1999 12:00 AM
Al Al2O3 1-Jun-1999 12:00 AM
Si SiO2 1-Jun-1999 12:00 AM
S FeS2 1-Jun-1999 12:00 AM
Cl KCl 1-Jun-1999 12:00 AM
K MAD-10 Feldspar 1-Jun-1999 12:00 AM
Ca Wollastonite 1-Jun-1999 12:00 AM
Zn Zn 1-Jun-1999 12:00 AM

Element	Weight%	Atomic%
N K	44.09	60.36
O K	13.55	16.24
Mg K	0.24	0.19
Al K	0.87	0.62
Si K	3.30	2.25
S K	28.18	16.85
Cl K	0.67	0.36
K K	0.28	0.14
Ca K	2.15	1.03
Zn K	6.67	1.96
Totals	100.00	

Figure S2. The EDAX analysis of PPS.

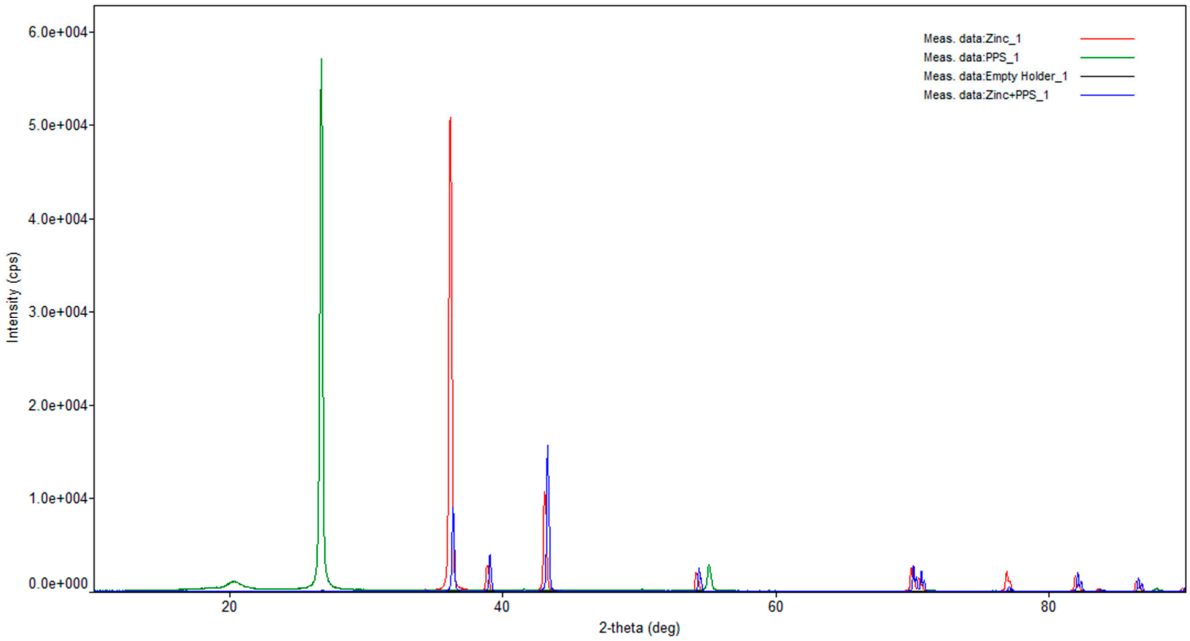

Figure S3. The XRD analysis of Zn_PPS.

Table S1 shows the relative permeability, permittivity, boundary conditions, mesh quality, and frequency range of coating materials.

Table S1. EMI simulation parameters of coatings.

Parameters/Sample	Zinc	PPS	PEEK	PPA	Al-Bronze
**Relative Permeability**	1.00021	1	1	1	1.000021
**Permittivity**	3.21	3.21	3.23	4.3	3.21
**Boundary conditions**	Perfect E	Perfect E	Perfect E	Perfect E	Perfect E
**Mesh quality**	Default	Default	Default	Default	Default
**Frequency (GHz)**	0.01 – 1.5 GHz	0.01 – 1.5 GHz	0.01 – 1.5 GHz	0.01 – 1.5 GHz	0.01-1.5 GHz
**Step frequency (MHz)**	7.45	7.45	7.45	7.45	7.45
**Excitation type**	Wave port	Wave port	Wave port	Wave port	Wave port

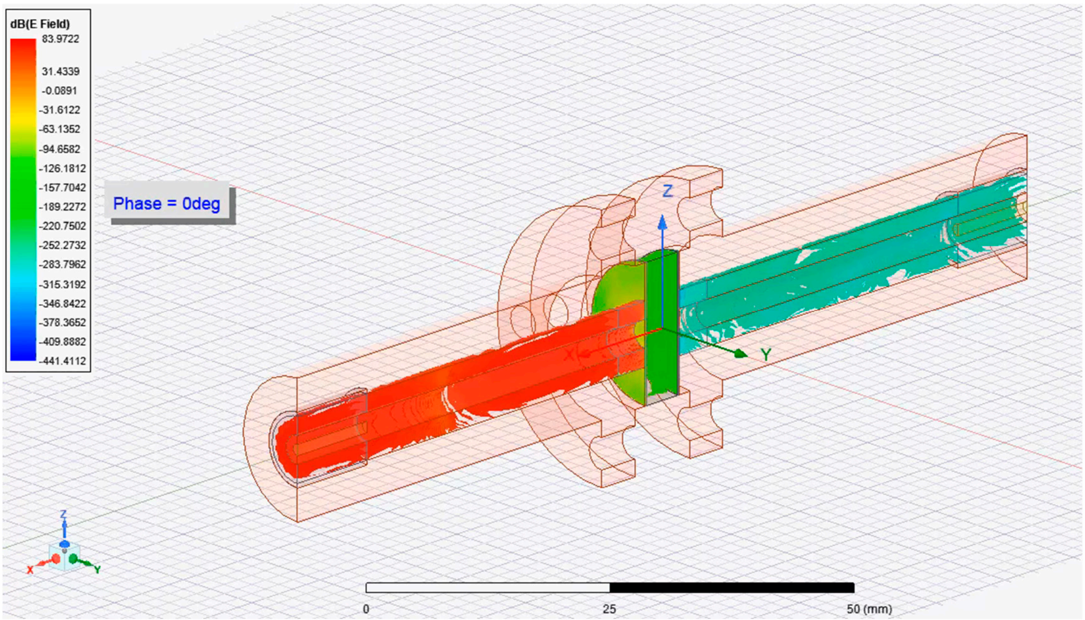

Figure S4. Simulation of E-Field at 1.05 GHz for PPA sample in the EMI fixture.
